# Mechanism and intervention of murine transfusion-related acute lung injury caused by anti-CD36 antibodies

**DOI:** 10.1172/jci.insight.165142

**Published:** 2023-03-22

**Authors:** Da-Wei Chen, Tian Kang, Xiu-Zhang Xu, Wen-Jie Xia, Xin Ye, Yong-Bin Wu, Yao-Ri Xu, Jing Liu, Hui Ren, Jing Deng, Yang-Kai Chen, Hao-Qiang Ding, Muhammad Aslam, Wioleta M. Zelek, B. Paul Morgan, Rick Kapur, Sentot Santoso, Yong-Shui Fu

**Affiliations:** 1 Institute of Blood Transfusion, Guangzhou Blood Centre, Guangzhou, Guangdong, China.; 2 Institute for Clinical Immunology and Transfusion Medicine, Justus Liebig University, Giessen, Germany.; 3 The First School of Clinical Medicine, Southern Medical University, Guangzhou, Guangdong, China.; 4 The Second Affiliated Hospital of Guangzhou Medical University, Guangzhou, Guangdong, China.; 5 Department of Cardiology and Angiology, Justus Liebig University, Giessen, Germany.; 6 Dementia Research Institute, School of Medicine, Cardiff University, Cardiff, United Kingdom.; 7 Department of Experimental Immunohematology, Sanquin Research and Landsteiner Laboratory, Amsterdam UMC, University of Amsterdam, Amsterdam, Netherlands.; 8 Department of Transfusion Medicine, School of Laboratory Medicine and Biotechnology, Southern Medical University, Guangzhou, Guangdong, China.

**Keywords:** Immunology, Pulmonology, Complement, Drug therapy, Monocytes

## Abstract

Anti-CD36 Abs have been suggested to induce transfusion-related acute lung injury (TRALI) upon blood transfusion, particularly in Asian populations. However, little is known about the pathological mechanism of anti-CD36 Ab–mediated TRALI, and potential therapies have not yet been identified. Here, we developed a murine model of anti-CD36 Ab–mediated TRALI to address these questions. Administration of mouse mAb against CD36 (mAb GZ1) or human anti-CD36 IgG, but not GZ1 F(ab′)_2_ fragments, induced severe TRALI in *Cd36^+/+^* male mice. Predepletion of recipient monocytes or complement, but not neutrophils or platelets, prevented the development of murine TRALI. Moreover, plasma C5a levels after TRALI induction by anti-CD36 Abs increased more than 3-fold, implying a critical role of complement C5 activation in the mechanism of Fc-dependent anti-CD36–mediated TRALI. Administration of GZ1 F(ab′)_2_, antioxidant (*N*-acetyl cysteine, NAC), or C5 blocker (mAb BB5.1) before TRALI induction completely protected mice from anti-CD36–mediated TRALI. Although no significant amelioration in TRALI was observed when mice were injected with GZ1 F(ab′)_2_ after TRALI induction, significant improvement was achieved when mice were treated postinduction with NAC or anti-C5. Importantly, anti-C5 treatment completely rescued mice from TRALI, suggesting the potential role of existing anti-C5 drugs in the treatment of patients with TRALI caused by anti-CD36.

## Graphical abstract

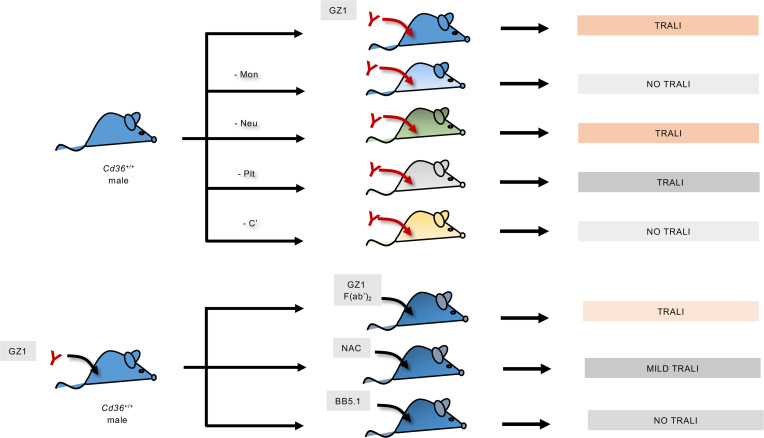

## Introduction

Transfusion-related acute lung injury (TRALI) is a life-threatening syndrome that occurs within 6 hours of a blood transfusion and is characterized by acute respiratory distress and the development of noncardiogenic pulmonary edema (1). The 2-hit model (2, 3) is the most widely accepted hypothesis: it suggests that TRALI is the result of 2 independent events that trigger a cascade of immune reactions leading to vascular endothelium damage, pulmonary capillary fluid leakage, and, consequently, lung edema (1, 4). Risk factors such as inflammation present in a patient can act as the first hit, whereas the second hit can be conveyed by transfusion-specific factors that can be divided into Ab- and non–Ab-mediated TRALI (1, 4–6). Abs against HLA class II and HNA-3 are the major clinical drivers of severe TRALI (7, 8).

In the classical Ab-mediated TRALI model, neutrophils have been implicated as important factors (9–11), whereas in some studies, authors have reported that monocytes and macrophages, rather than neutrophils, play crucial roles in the development of TRALI (12, 13). Significant controversy, however, still exists concerning the role of platelets in TRALI (14). Recent studies in murine models have demonstrated that osteopontin, secreted by macrophages, was critically required for TRALI induction via stimulation of neutrophil recruitment into the lungs (15) and that Fc-mediated complement activation was an important feature of TRALI (16).

Individuals lacking CD36 on platelets and on monocytes (type I deficiency) generate anti-CD36 Abs (hereafter referred to as anti-CD36) due to immunization after blood transfusion or during pregnancy. Type I CD36 deficiency is extremely rare in White people but is found at relatively high frequency among Asians (>0.5%) (17, 18). In Asians, anti-CD36 plays a critical role in the pathological mechanism of platelet transfusion refractoriness and fetal/neonatal alloimmune thrombocytopenia (FNAIT) (19–22).

In Japan, TRALI cases caused by anti-CD36 have been reported (23, 24); incubation of monocytes with anti-CD36 plasma derived from a female blood donor that induced TRALI in patients resulted in the production of the pro-inflammatory mediators LTB_4_ and TNF-α (23). This reaction was dependent on the presence of human lung microvascular endothelial cells (HLMVECs), indicating the role of cellular crosstalk between monocytes and lung endothelial cells. Nevertheless, the exact mechanism of anti-CD36–mediated TRALI remains unknown.

Although TRALI is a leading cause of transfusion-related death, specific treatments are currently unavailable (1, 4). Hence, supportive measures remain the mainstay for patients with TRALI (25). In recent years, animal models have been established to study the mechanism of TRALI and to evaluate specific treatments. Some drugs, including aspirin, intravenous immunoglobulin (IVIG), anti-osteopontin Ab, and IL-10, have been shown to prevent TRALI in a mouse model of anti–MHC class I–mediated TRALI (10, 15, 26, 27). However, no promising preclinical approaches have been reported for other Ab specificities.

In this study, we developed a murine model to study the pathomechanism of anti-CD36–mediated TRALI and to subsequently test different possible agents for efficacy in the treatment of severe TRALI caused by anti-CD36 Abs.

## Results

### Mouse and human anti-CD36 Abs induce severe TRALI in a 2-hit mouse model.

**Figure 1:**
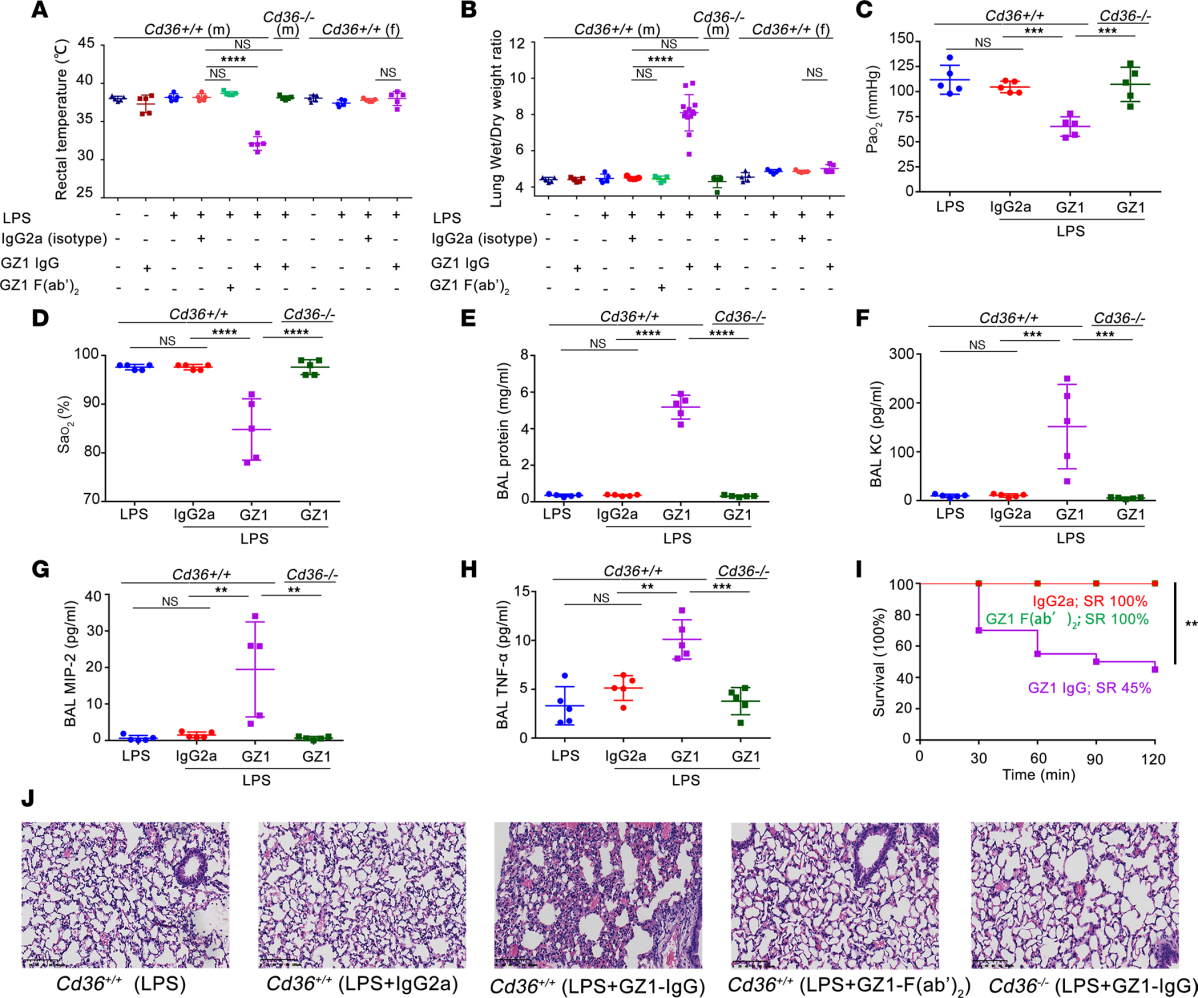
MAb GZ1 against CD36 induces TRALI in *Cd36^+/+^* male mice, not in female mice or *Cd36^–/–^* male mice. (**A**) Rectal temperatures (*n* = 5 in each group) and (**B**) lung W/D weight ratios of mice first untreated (-) or treated (+) with LPS, then mAb GZ1 IgG, mAb GZ1 F(ab′)_2_ or IgG2a isotype control were administered (*n* = 5 in each group; *n* = 13 in LPS pretreated *Cd36^+/+^* male with GZ1 injection). (**C**) Partial pressure of arterial oxygen (Pao_2_) (*n* = 5); (**D**) percentage of arterial oxygen (Sao_2_; %) (*n* = 5); concentration of (**E**) protein (*n* = 5); (**F**) KC (*n* = 5); (**G**) MIP-2 (*n* = 5); and (**H**) TNF-α (*n* = 5) in BAL of LPS-pretreated *Cd36^+/+^* male mice that were treated with GZ1 or IgG2a as described above. (**I**) SRs of LPS-pretreated *Cd36^+/+^* male mice injected with mAb GZ1 IgG (*n* = 20), mAb GZ1 F(ab′)_2_ (*n* = 10), or IgG2a isotype control (*n* = 10) were analyzed. (**J**) Histology performed on lung tissue from the indicated mouse groups. *Cd36^–/–^* male mice treated with mAb GZ1 were used as controls. Lung tissue sections were stained with H&E, and images were taken at ×20 original magnification. Representative images from each indicated group are shown. Scale bars: 100 μm. Statistical analysis was performed with 1-way ANOVA with Bonferroni’s correction for multiple comparisons (**A**–**H**) or with log-rank test (**I**). Each dot represents 1 mouse and error bars represent the SD. **P < 0.01, ***P < 0.001, ****P < 0.0001.

To establish a TRALI model in mice, mAb GZ1 (IgG2a; 0.4 mg/kg BW) was first administered i.v. to naive *Cd36^+/+^* male mice. No significant changes in rectal temperature or lung wet/dry (W/D) weight ratios were observed (Figure 1, A and B), suggesting that anti-CD36 alone could not induce TRALI. However, a significant decrease in rectal temperature (GZ1- vs. isotype-treated: 32.12°C ± 0.89°C vs. 38.16°C ± 0.47°C; *P* < 0.0001) (Figure 1A) and increase in lung W/D weight ratios (GZ1- vs. isotype-treated: 8.10 ± 1.00 vs. 4.50 ± 0.08; *P* < 0.0001) (Figure 1B) were detected when *Cd36^+/+^* male mice were pretreated with a low dose of LPS (0.1 mg/kg administered i.p.) and subsequently injected with mAb GZ1 (i.e., the 2-hit model), when compared with isotype controls. This phenomenon was not detected in *Cd36^–/–^* male mice. Administration of LPS alone in male and female *Cd36^+/+^* mice did not increase lung W/D weight ratios.

Interestingly, LPS-pretreated *Cd36^+/+^* female mice did not have elevated lung W/D weight ratios after injection with mAb GZ1, compared with the isotype controls (5.02 ± 0.22 vs. 4.86 ± 0.06, respectively; *P* > 0.05) (Figure 1B). In subsequent experiments, only LPS-pretreated *Cd36^+/+^* male mice receiving mAb GZ1 were analyzed (i.e., the 2-hit model).

Additional parameters characteristic of TRALI were observed in mAb GZ1–treated *Cd36^+/+^* male mice compared with isotype controls, including decreased partial pressure of oxygen (Pao_2_) (65.20 ± 9.73 mmHg vs. 104.60 ± 5.77 mmHg, respectively; *P* < 0.001) and oxygen saturation (Sao_2_) (84.80% ± 6.30% vs. 97.60% ± 0.55%, respectively; *P* < 0.0001) (Figure 1, C and D). Increased protein (5175.84 ± 654.12 vs. 357.82 ± 40.70 μg/mL, respectively; *P* < 0.0001), chemokine (keratinocyte-derived chemokine [KC]: 151.51 ± 86.39 pg/mL vs. 10.44 ± 3.43 pg/mL, respectively, *P* < 0.001; macrophage inflammatory protein 2 [MIP-2]: 19.45 ± 13.00 pg/mL vs. 1.52 ± 0.79 pg/mL, respectively, *P* < 0.01), and cytokine (TNF-α: 10.10 ± 2.00 pg/mL vs. 5.13 ± 1.27 pg/mL, respectively; *P* < 0.01) concentrations were also detected in bronchoalveolar lavage (BAL) fluid from mAb GZ1–treated mice when compared with isotype controls (Figure 1, E–H).

Survival rate (SR) analysis using Kaplan-Meier curves showed that more than half of the mice did not survive beyond 2 hours after administration of mAb GZ1 IgG, compared with 100% survival in isotype or mAb GZ1 F(ab′)_2_–treated cohorts (SR: 45% vs. 100%; *P* < 0.01) (Figure 1I). Pathological analysis of the lungs showed the presence of pulmonary edema and alveolar septal thickening in male mice primed with LPS and treated with mAb GZ1 IgG but not with F(ab′)_2_ fragments. In *Cd36^–/–^* male mice, no pathological change was observed (Figure 1J).

**Figure 2:**
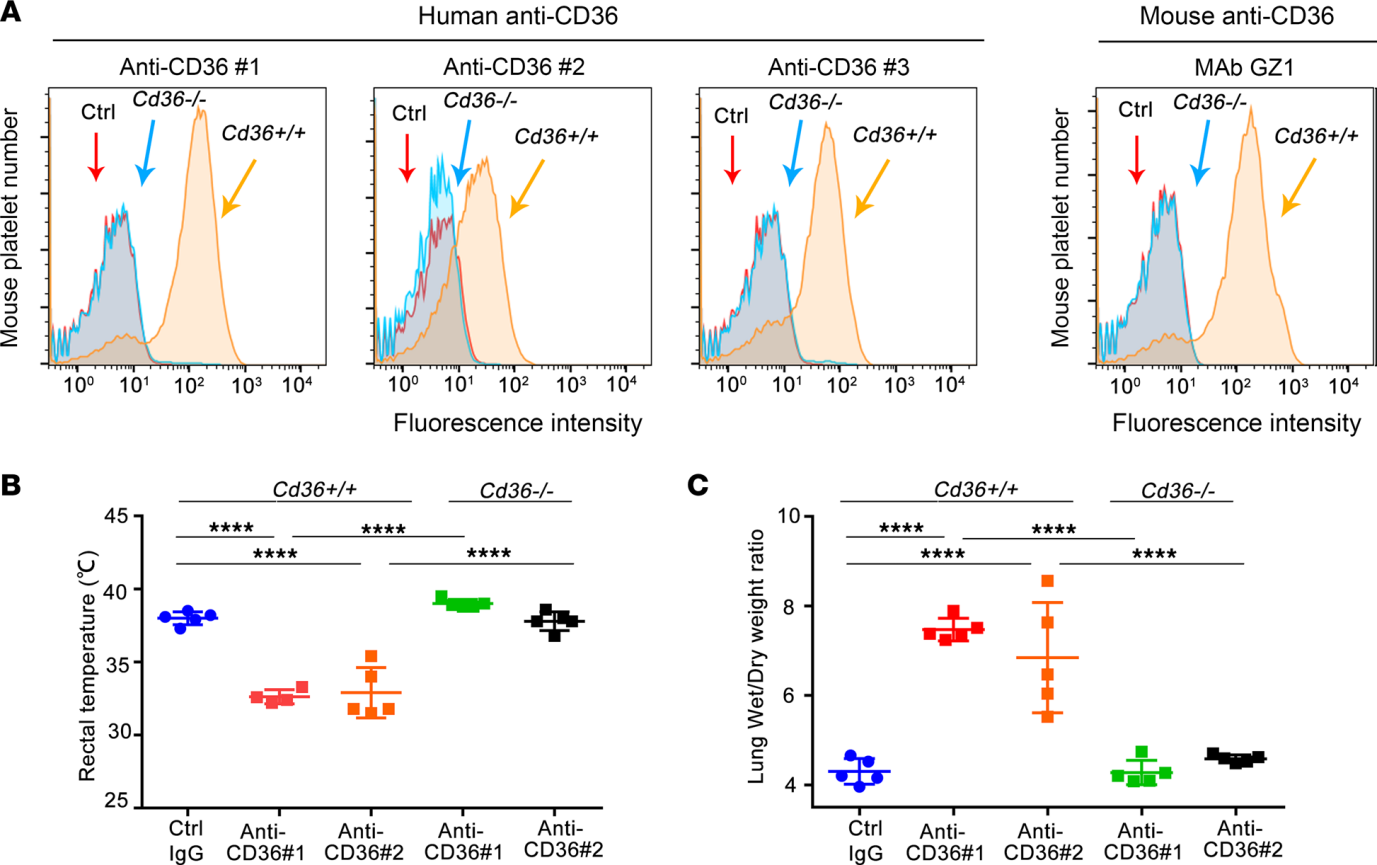
Purified human anti-CD36 IgG induces TRALI in *Cd36^+/+^* male mice, not in *Cd36^–/–^* male mice. (**A**) Flow cytometric analysis of mAb GZ1 and human anti-CD36 sera 1, 2, and 3 samples incubated with platelets from *Cd36^+/+^* and *Cd36^–/–^* male mice as indicated (arrows). (**B**) Rectal temperatures and (**C**) lung W/D weight ratios of LPS-pretreated *Cd36^+/+^* or *Cd36^–/–^* male mice that were injected with purified IgG isolated from human normal AB serum (control IgG), anti-CD36 1, and anti-CD36 2. (**B** and **C**) Statistical analysis was performed with 1-way ANOVA with Bonferroni’s correction for multiple comparisons. Each dot represents 1 mouse (*n* = 5 in each group; *n* = 4 in rectal temperatures from anti-CD36 1 [1 mouse died in 27 minutes]) and error bars represent the SD. ****P < 0.0001.

To test the effects of human anti-CD36 Abs in this animal model, 3 sera samples from transfusion-immunized patients (anti-CD36 patients 1, 2, and 3 [hereafter, anti-CD36 1, 2, and 3, respectively]) were tested for binding to mouse platelets by flow cytometry. Ab binding was observed with *Cd36^+/+^* but not *Cd36^–/–^* mouse platelets, similar to the result with mAb GZ1 (Figure 2A). When purified IgG from anti-CD36 1 and 2 sera samples was injected into LPS-primed *Cd36^+/+^* mice, increases in lung W/D weight ratios and decreases in rectal temperatures were observed (Figure 2, B and C). These reactions were not observed in *Cd36^–/–^* mice. Unfortunately, anti-CD36 3 serum could not be tested in this model, because of limited materials. These results demonstrated that both murine and human anti-CD36 Abs can induce severe TRALI in male, but not female, mice in a 2-hit model.

### Anti-CD36–induced TRALI depends on Fcγ receptors.

Interestingly, injection of mAb GZ1 F(ab′)_2_ fragment did not lead to the development of TRALI in our model. Decreased rectal temperature (GZ1- vs. isotype-treated: 38.68°C ± 0.25°C vs. 38.16°C ± 0.47°C; *P* > 0.05) and increased lung W/D weight ratio (GZ1- vs. isotype-treated: 4.45 ± 0.16 vs. 4.50 ± 0.08; *P* > 0.05) were not observed, compared with the isotype control (Figure 1, A and B). Similar observations were found when mice were treated prophylactically with IVIG or with mAb 2.4G2 against CD16/CD32 before TRALI induction with anti-CD36 (Supplemental Figure 1; supplemental material available online with this article; https://doi.org/10.1172/jci.insight.165142DS1).

### Monocytes and complement are important pathogenic factors for murine TRALI induced by anti-CD36.

To assess the contributions of different recipient blood cell types and complement on the mechanism of anti-CD36–mediated TRALI, monocytes, neutrophils, platelets, and complement were depleted in vivo before TRALI induction with mAb GZ1 (Supplemental Figure 2).

**Figure 3:**
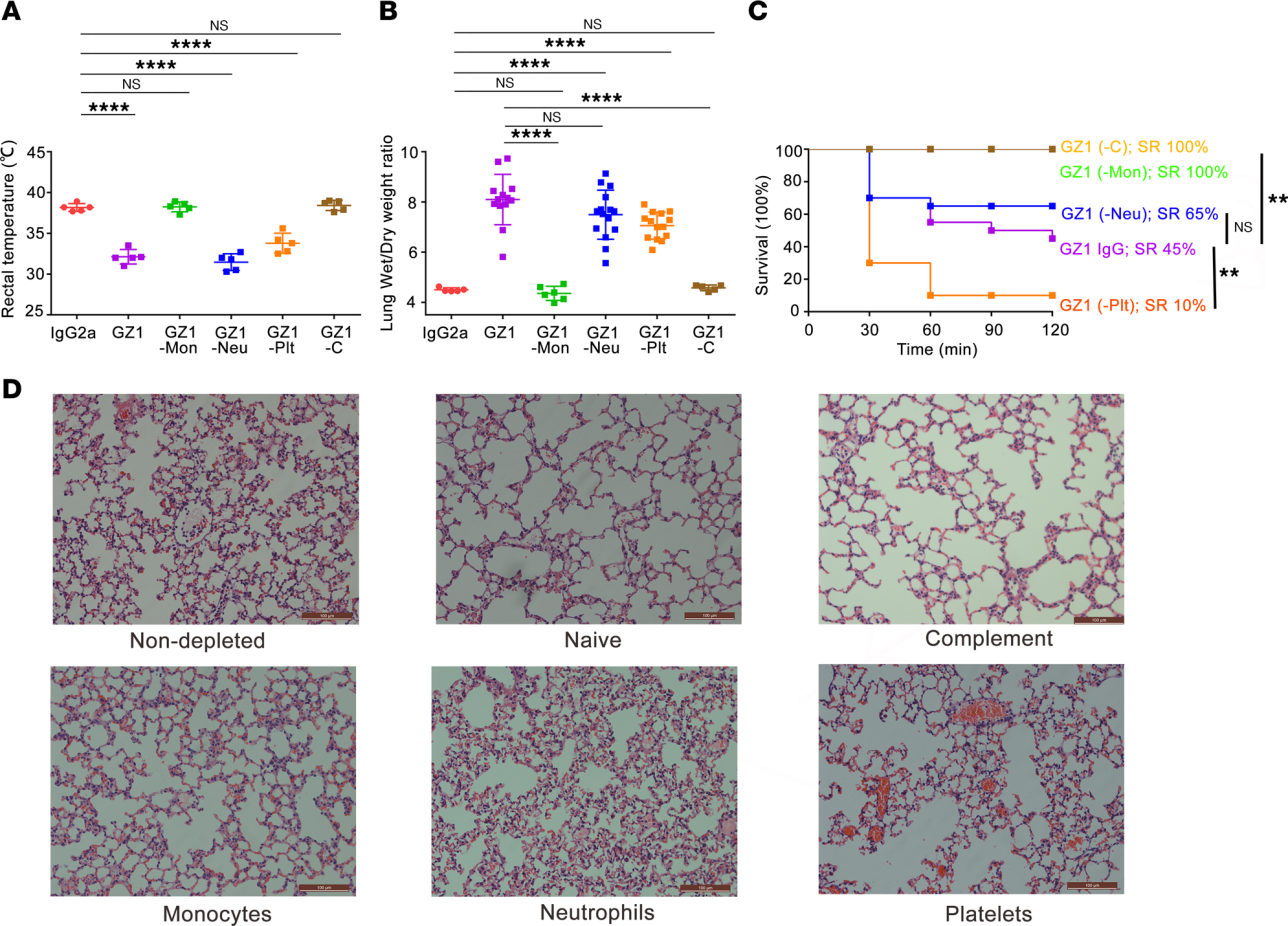
Monocyte depletion and complement depletion protect mice from TRALI induced by mAb GZ1 against CD36. (**A**) Rectal temperatures (*n* = 5 in each group) and (**B**) lung W/D weight ratios of LPS-pretreated *Cd36^+/+^* male mice that were injected with mAb GZ1 after depletion of monocytes (-Mon; *n* = 6), neutrophils (-Neu; *n* = 15), platelets (-Plt; *n* = 14) and complement (-C; *n* = 5). (**C**) SRs of depleted mice injected with mAb GZ1 were analyzed (*n* = 10 in monocytes and complement depletion group; *n* = 20 in neutrophils and platelets depletion group). (**D**) Histology performed on lung tissue from the indicated mouse groups. Lung tissue sections were stained with H&E, and images were taken at ×20 original magnification. Representative images from each indicated group are shown. Scale bars: 100 μm. Statistical analysis was performed with 1-way ANOVA with Bonferroni’s correction for multiple comparisons (**A** and **B**) or with log-rank test (**C**). Each dot represents 1 mouse and error bars represent the SD. **P < 0.01, ****P < 0.0001.

In our 2-hit model, rectal temperatures dropped significantly in neutrophil- and platelet-depleted mice at 30 minutes after TRALI induction, similar to nondepleted mice; in contrast, neither monocyte- nor complement-depleted mice had a change in rectal temperature (Figure 3A). Increased lung W/D weight ratios were not observed in monocyte-depleted (4.36 ± 0.28; *P* > 0.05) and complement-depleted mice (4.58 ± 0.11; *P* > 0.05) when compared with isotype controls (4.50 ± 0.08), whereas neutrophil-depleted mice had increased lung W/D weight ratios comparable to those of isotype controls (7.49 ± 0.98 vs. 4.50 ± 0.08; *P* < 0.0001) (Figure 3B). Depletion of platelets with anti–GPIb-α before TRALI induction led to significant increases in lung W/D weight ratios in comparison with isotype controls (7.06 ± 0.54 vs. 4.50 ± 0.08, respectively; *P* < 0.0001) (Figure 3B).

All monocyte- and complement-depleted mice survived 2 hours after TRALI induction (SR, 100%) (Figure 3C). In contrast, the SR of neutrophil-depleted mice at 2 hours was not significantly different from that of nondepleted mice (65% vs. 45%; *P* > 0.05) (Figure 3C). Interestingly, even lower SRs were observed in platelet-depleted mice compared with nondepleted mice (10% vs. 45%; *P* < 0.01) (Figure 3C), likely due to abundant hemorrhages found in the lung alveoli, indicating the protective role of platelets in anti-CD36–mediated TRALI, most probably by maintaining vascular integrity.

Lung histological analysis of neutrophil- and platelet-depleted mice revealed fibrin deposition (28) and interalveolar capillary hyperemia resembling that observed in nondepleted TRALI mice. As noted above, hemorrhages in the alveoli were found in platelet-depleted mice. In contrast, no histological signs of acute lung injury were observed in monocyte- or complement-depleted mice; lung histology resembled that observed in naive mice (Figure 3D).

### Anti-CD36–mediated murine TRALI is associated with increased plasma C5a levels.

**Figure 4:**
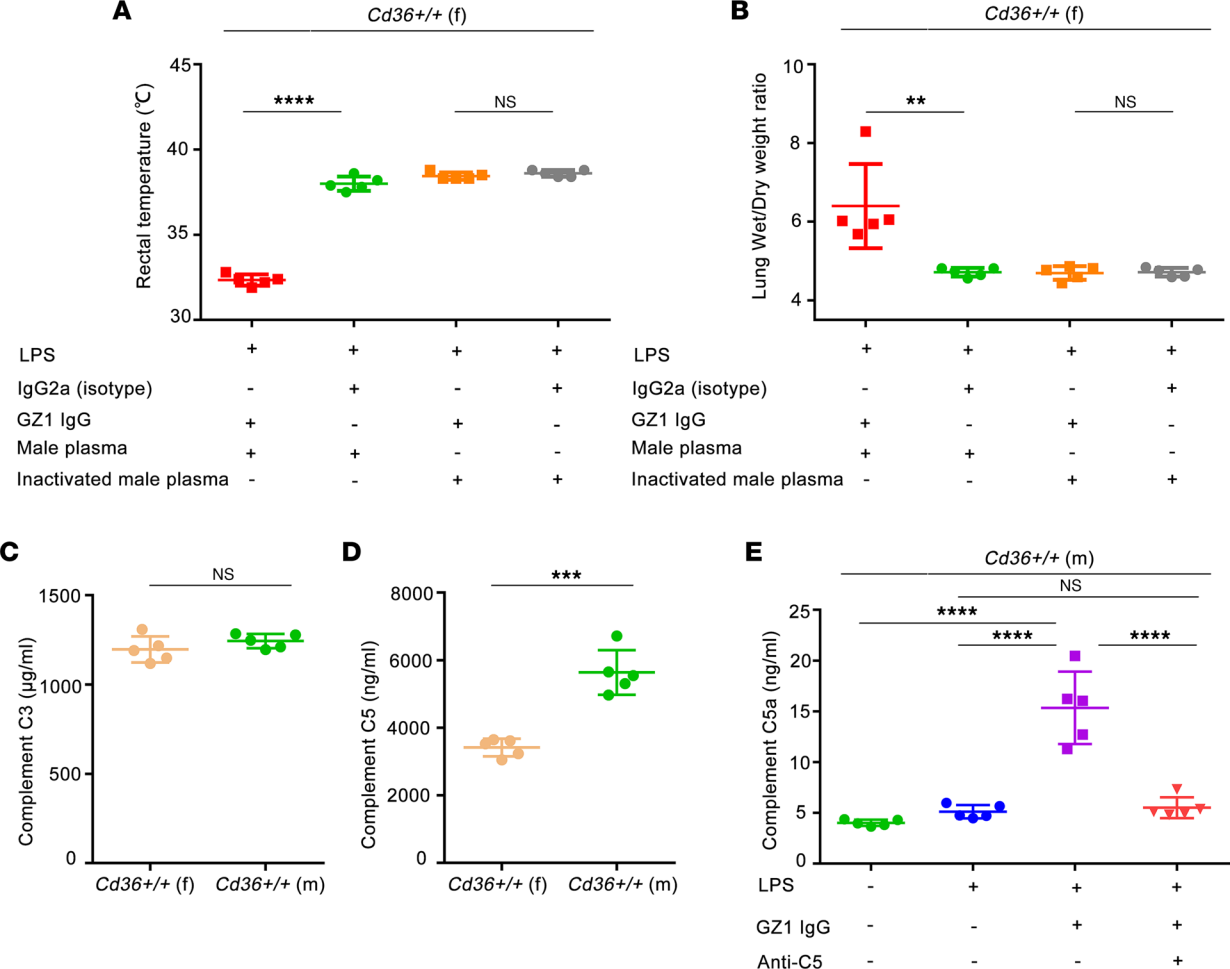
Female mice can develop TRALI after transfusion with plasma from male mice. (**A**) Rectal temperatures and (**B**) lung W/D weight ratios of LPS-pretreated *Cd36^+/+^* female mice were injected with mAb GZ1 after transfusion with pooled male plasma or inactivated plasma. IgG2a isotype was treated as the control. The concentration of C3 (**C**) and C5 (**D**) in plasma of untreated *Cd36^+/+^* female and male mice. (**E**) Concentration of C5a in nontreated, LPS-treated, GZ1-treated, and GZ1 plus anti-C5–treated *Cd36^+/+^* male mice. Statistical analysis was performed with 1-way ANOVA with Bonferroni’s correction for multiple comparisons (**A**, **B**, and **E**) or with a 2-tailed unpaired Student’s *t* test (**C** and **D**). Each dot represents 1 mouse (*n* = 5 in each group) and error bars represent the SD. **P < 0.01, ***P < 0.001, ****P < 0.0001.

In this study, strong TRALI was observed in male, but not in female, mice. However, female mice injected with GZ1 could also develop TRALI after transfusion with plasma from male mice, with significantly decreased rectal temperature (GZ1- vs. isotype-treated: 32.34°C ± 0.33°C vs. 38.00°C ± 0.42°C; *P* < 0.0001) (Figure 4A) and increased lung W/D weight ratio (GZ1- vs. isotype-treated: 6.40 ± 1.07 vs. 4.71 ± 0.11; *P* < 0.01) (Figure 4B), compared with isotype control, and increased protein in BAL, documented by the lung histology (Supplemental Figure 3, A and B). In contrast, TRALI did not occur when female mice were transfused with heat-inactivated male plasma (Figure 4, A and B), indicating that female mice possess lower complement level than do male mice (29).

Similar plasma C3 levels were found in both sexes (female vs. male mice: 1,196.92 ± 73.15 μg/mL vs. 1,243.72 ± 39.71 μg/mL; *P* > 0.05) (Figure 4C). However, our female mice had significantly lower plasma C5 concentrations when compared with male mice (female vs. male mice: 3,415.55 ± 259.86 ng/mL vs. 5,637.58 ± 658.78 ng/mL, respectively; *P* < 0.001) (Figure 4D). Significantly lower C5 concentrations were also found in complement-depleted male mice when compared with the nondepleted group (complement-depleted vs. nondepleted mice: 743.03 ± 546.36 ng/mL vs. 5,637.58 ± 658.78 ng/mL, respectively; *P* < 0.0001) (see also Supplemental Figure 2E).

Interestingly, significantly higher plasma C5a levels (15.35 ± 3.56 ng/mL) were detected after TRALI induction with mAb GZ1 than with LPS alone (5.11 ± 0.66 ng/mL; *P* < 0.0001) and compared with naive controls (4.02 ± 0.37 ng/mL; *P* < 0.0001) (Figure 4E). C5a levels did not increase when mice were pretreated with neutralizing mAb BB5.1 against C5 prior to TRALI induction with mAb GZ1 (pretreated with BB5.1 vs. LPS alone: 5.51 ± 1.03 ng/mL vs. 5.11 ± 0.66 ng/mL; *P* > 0.05) (Figure 4E) (30).

### Human monocytes primed with human anti-CD36 sera impair human pulmonary endothelial cell permeability.

**Figure 5:**
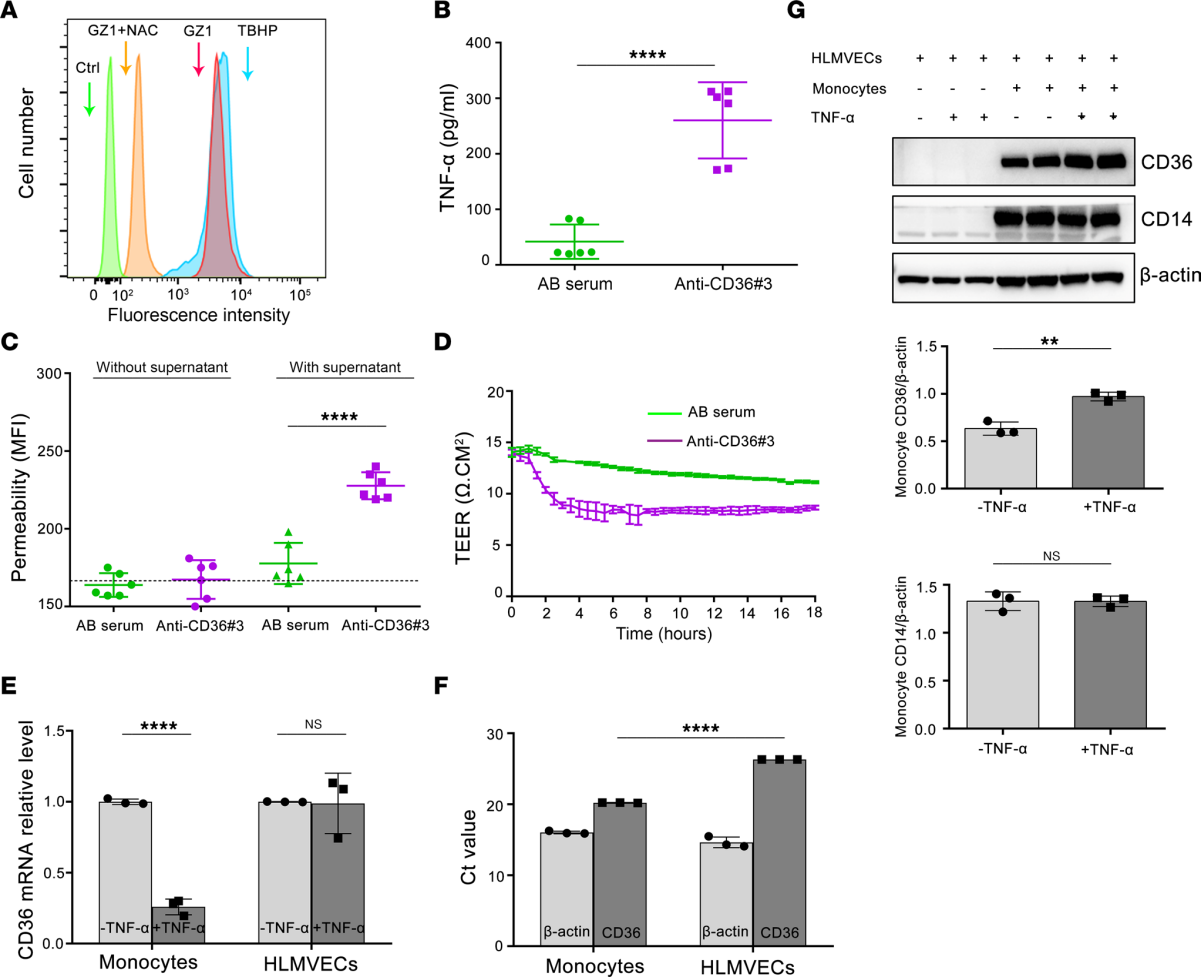
Cytokines derived from PBMCs treated with anti-CD36 Abs increase HLMVECs’ permeability. (**A**) Flow cytometric analysis of mAb GZ1–induced ROS generation in monocytes. Tert-butyl hydroperoxide–treated (TBHP) and PBS-treated monocytes (Ctrl) were used as positive and negative controls, respectively. Monocytes were gated using side scatter versus forward scatter plot and analyzed by flow cytometry. A representative result of 4 independent experiments is shown. (**B**) The concentration of TNF-α in cell supernatant derived from PBMCs incubated with anti-CD36 3 or AB serum. (**C**) Cytokines derived from PBMCs treated with anti-CD36 3 increased HLMVEC permeability. Results are expressed as mean ± SD of duplicates from 3 independent experiments. The dotted line showed fluorescence intensity of HLMVEC monolayers incubated with culture medium. (**D**) Cytokines derived from PBMCs treated with anti-CD36 3 decreased TEER. TEER was measured using a real-time program by cellZscope in duplicates from 2 independent experiments. (**E**) TNF-α decreased CD36 mRNA expression on monocytes, not on HLMVECs. CD36 mRNA expression was quantified using RT-PCR and normalized to β-actin. Data represent the mean ± SD of the relative quantification of CD36 mRNA expression measured in 3 experiments. (**F**) Ct values of untreated monocyte and HLMVEC in RT-PCR, and β-actin was run as a control. Data represent the mean ± SD of 3 experiments. (**G**) Western blots showed the upregulation of CD36 expression on monocytes adherent to TNF-α–treated HLMVECs. CD36, CD14, and β-actin bands were quantified using Image Lab software and represented as CD36/β-actin and CD14/β-actin. Representative images from 3 independent experiments are presented. Statistical analysis was performed with a 2-tailed unpaired Student’s *t* test (**B**, **E**, and **G**), or with 1-way ANOVA with Bonferroni’s correction for multiple comparisons (**C** and **F**). **P < 0.01, ****P < 0.0001.

Next, we asked whether anti-CD36 could induce monocyte activation and thereby alter endothelial permeability. First, we found that binding of mAb GZ1 onto monocytes caused ROS generation that could be inhibited by antioxidant *N*-acetyl cysteine (NAC) (Figure 5A). Furthermore, analysis of supernatant from monocytes after treatment with anti-CD36 3 serum from a patient with TRALI showed significantly higher TNF-α concentrations compared with monocytes treated with control serum (260.13 ± 68.63 pg/mL vs. 41.79 ± 30.94 pg/mL, respectively; *P* < 0.0001) (Figure 5B). Treatment of HLMVECs with the supernatant increased transendothelial permeability, as shown by higher influx of fluorescently labeled BSA through the HLMVEC monolayer in the Transwell assay in comparison with HLMVECs treated with control supernatant (MFI: 227.67 ± 8.69 vs. 177.67 ± 13.22, respectively; *P* < 0.0001) (Figure 5C). This phenomenon was not found when HLMVECs were treated with anti-CD36 3 serum directly. These findings were confirmed by transendothelial resistance (TEER) analysis: HLMVECs treated with supernatant from monocytes primed with anti-CD36 3 had lower TEER than did HLMVECs treated with control serum (Figure 5D).

### Upregulation of CD36 expression on monocytes after adhesion on TNF-α–activated endothelial cells.

Subsequently, we measured the effect of TNF-α on CD36 expression by quantitative reverse transcription PCR. Significant downregulation of CD36 expression was found on monocytes, but not on HLMVECs, after TNF-α treatment (Figure 5E). Real-time quantitative PCR showed that monocytes expressed significantly more CD36 transcript than did HLMVECs (26.30 vs. 22.22, respectively; *P* < 0.0001) (Figure 5F). When monocytes were co-cultured with TNF-α–treated HLMVECs, significant upregulation of CD36 expression, compared with monocytes that had adhered to untreated HLMVECs, was observed, as shown by immunoblotting (0.97 vs. 0.63, respectively; *P* < 0.01) (Figure 5G). In a control experiment, no change in CD14 expression on monocytes was found.

### Inhibition of anti-CD36–mediated murine TRALI.

In the following experiments, the 2-hit TRALI model was induced by mAb GZ1, as described above. The inhibitors mAb GZ1 F(ab′)_2_, antioxidant NAC, and anti-C5 (mAb BB5.1) were administered either before (before TRALI: prophylactic) or after (after TRALI: treatment) TRALI induction.

**Figure 6:**
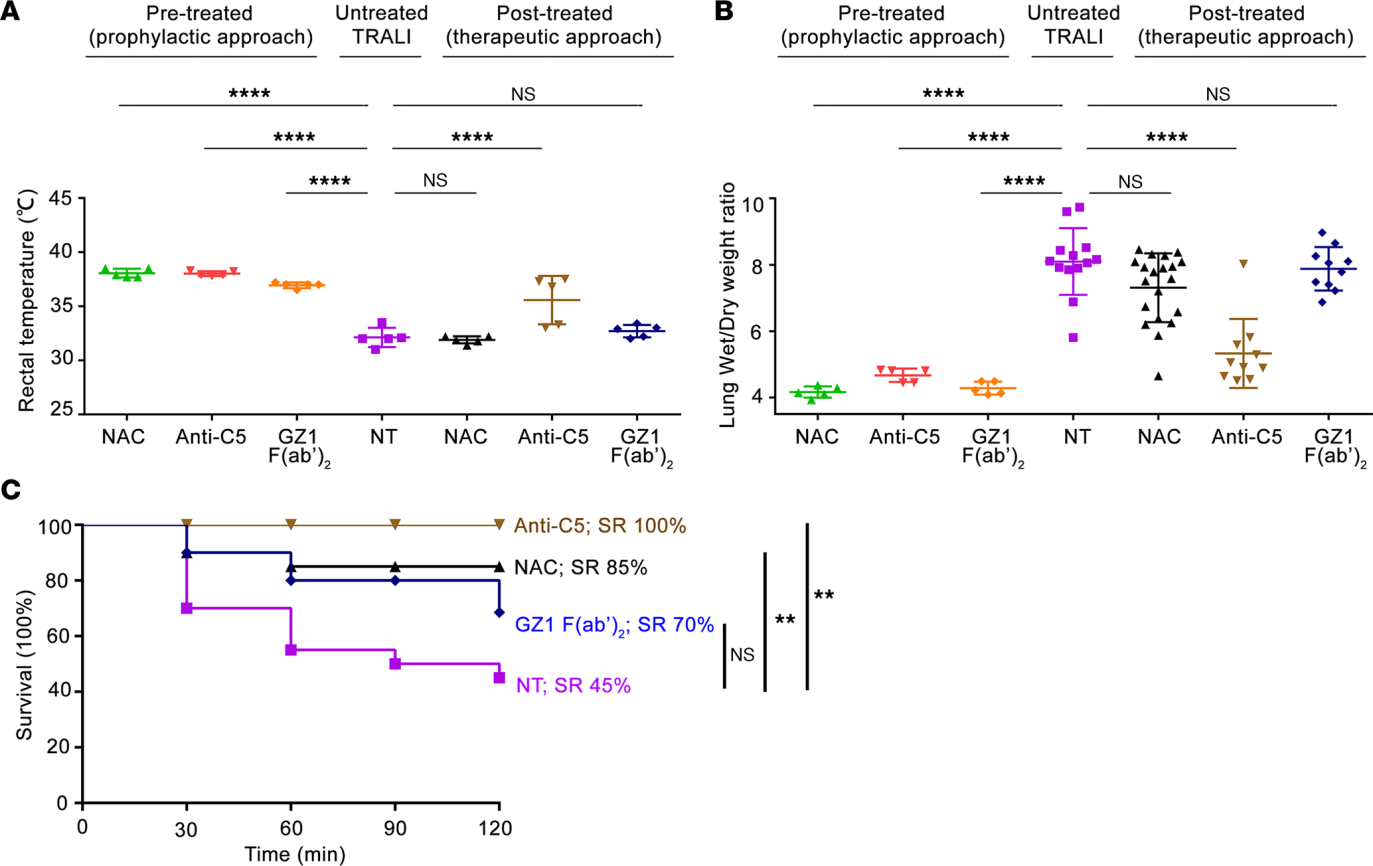
Anti-C5 can not only prevent but also completely rescue mice from anti-CD36–mediated TRALI. In the prophylactic approach, GZ1 F(ab′)_2_ (*n* = 5), NAC (*n* = 5), or anti-C5 (*n* = 5) was used as pretreatment before TRALI induction with mAb GZ1. In the therapeutic approach, TRALI was first induced with mAb GZ1. Mice were treated with GZ1 F(ab′)_2_ (*n* = 10), NAC (*n* = 20), or anti-C5 (*n* = 10) after TRALI. Untreated TRALI mice (NT) were treated as control (Ctrl). (**A**) Rectal temperatures and (**B**) lung W/D weight ratios were measured as described above. (**C**) SRs of *Cd36^+/+^* male mice treated with different inhibitors after TRALI induction were analyzed. Statistical analysis was performed with 1-way ANOVA with Bonferroni’s correction for multiple comparisons (**A** and **B**) or with log-rank test (**C**). Each dot represents 1 mouse and error bars represent the SD. **P < 0.01, ****P < 0.0001.

Administration of each inhibitor before TRALI prevented the development of TRALI; neither significant decrease of rectal temperature nor significant increase in lung W/D weight ratios was observed when compared with mice that did not receive these inhibitors (Figure 6, A and B). In contrast, administration of GZ1 F(ab′)_2_ after TRALI did not prevent TRALI. No significant difference in the lung W/D weight ratios between TRALI mice receiving GZ1 F(ab′)_2_ and untreated TRALI mice was found (7.88 ± 0.65 vs. 8.10 ± 1.00; *P* > 0.05) (Figure 6B). Significant improvement was detected when injected NAC was administered together with inhalation of atomized NAC. Although the lung W/D weight ratios were not significantly lower than in the untreated TRALI cohort (NAC vs. untreated: 7.31 ± 1.04 vs. 8.10 ± 1.00; *P* >0.05) (Figure 6B), the survival of mice treated with NAC after TRALI significantly improved (85% vs. 45%, respectively; *P* < 0.01). No significant improvement was achieved with NAC solution alone (data not shown). This improvement in lung W/D weight ratios became more significant when mAb BB5.1 was administered after TRALI compared with the untreated TRALI group (5.33 ± 1.04 vs. 8.10 ± 1.00, respectively; *P* < 0.0001) (Figure 6B). Accordingly, all mice survived after induction treatment with mAb BB5.1 (SR, 100%), but only 85% and 70% survived after treatment with NAC and GZ1 F(ab′)_2_, respectively (Figure 6C).

## Discussion

In this study, we developed a murine model of anti-CD36–induced TRALI and found that murine mAbs and human Abs against CD36 induced severe TRALI in *Cd36^+/+^* male mice, but not in *Cd36^+/+^* female and *Cd36^–/–^* male mice. This TRALI response was only observed in the presence of a low dose of LPS (i.e., the 2-hit model) (31) and was characterized by increased lung W/D weight ratios, decreased rectal temperatures, decreased Pao_2_ and Sao_2_, and increased protein, chemokine (KC, MIP-2), and cytokine (TNF-α) concentrations in BAL. This reaction is not only dependent on the sex of the mice but also on the Fc part of the Abs, the presence of monocytes, ROS generation by monocytes, TNF-α production, and C5 complement activation. That anti-CD36 from human sera could also induce TRALI in our murine model is likely due to the high degree of homology (approximately 85%) between the human and mouse CD36 protein (32) and because most CD36 Abs react with immunodominant epitopes (20, 33).

Meanwhile, the contribution of different cell types to the pathogenesis of TRALI has become increasingly clear (1). It is commonly believed that Ab-mediated TRALI is the result of pulmonary damage caused by neutrophils. However, the pathogenic role of other cells, including monocytes, macrophages, endothelial cells, and platelets, has been well studied (1, 14, 15, 34–37). Nevertheless, these cellular pathways remain complex and somehow depend on Ab specificities (1).

Here, we found that anti-CD36 could still induce TRALI in the absence of neutrophils. In other TRALI models, neutrophil depletion completely protected mice from anti–MHC class I–mediated TRALI (10). This discrepancy is most probably due to the fact that neutrophils do not express CD36 antigens (38). The SR of neutrophil-depleted mice, however, was higher when compared with that of nondepleted mice (SR, 65% vs. 45%, respectively), indicating that neutrophils do contribute to the severity of TRALI. In a previous study, researchers demonstrated HLA-DR is inducible in human neutrophils both in vitro and in vivo by IFN-γ or GM-CSF (39), suggesting that neutrophils may play a more important role in anti-HLA class II–mediated TRALI (40). Whether this is also true for CD36 is currently unknown.

Several studies have shown that monocytes are indispensable for the mechanism of Ab-mediated TRALI (12, 15, 41). Sachs et al. (42) showed that Abs against MHC class II antigens, which are expressed on monocytes but not on neutrophils, triggered TRALI in an ex vivo rat model. In anti–MHC class I murine models, depletion/inactivation of monocytes/macrophages completely suppressed TRALI (12, 13, 15, 41). Here, we showed that depletion of monocytes completely abolished anti-CD36–induced TRALI. Normal lung W/D weight ratios and rectal temperatures were detected in monocyte-depleted mice. All mice survived (SR, 100%), indicating a pivotal role for monocytes in anti-CD36–mediated murine TRALI.

It has been demonstrated that gp91phox KO mice are completely protected from TRALI induced by anti–MHC class I Abs, suggesting that the formation of ROS is important for the development of TRALI (10). An additional study in mice showed the important role of CD36 in H_2_O_2_-induced lung injury (43). We found that anti-CD36 could induce ROS production, which could be inhibited by the antioxidant NAC, both in vitro and in vivo.

The role of platelets in TRALI has been less well defined (14, 36). Some studies have found recipient platelets to be pathogenic (26, 44), whereas other studies have found them to be dispensable for the onset of TRALI (12, 45). This discrepancy may have been caused by different factors, including different experimental methodologies. In our murine model, platelet depletion was induced by anti–GPIb-α administration, and this depletion aggravated the severity of anti-CD36–induced TRALI: SR of the mice in this cohort (10%) was much lower than that of the controls. Besides signs of TRALI, bleeding tendency was observed in our mice, indicating that platelets may maintain vascular integrity in anti-CD36–mediated TRALI (46).

Furthermore, we found that F(ab′)_2_ fragment of anti-CD36 could not induce TRALI, indicating the role of Fcγ receptors (FcγRs). In a previous study, researchers demonstrated that Fc block (mAb 2.4G2) against CD16/CD32 could prevent anti-MHC Ab–induced TRALI (12). Similarly, pretreatment with mAb 2.4G2 prevented the development of TRALI in our mice, a finding that strengthens the idea of a role of FcγR in anti-CD36–mediated TRALI. Along this line, prophylactic injection of IVIG also prevented mice’s TRALI development (47). The question of whether activating or inhibitory FcγR is involved is intriguing and needs further investigation (48).

Our previous study demonstrated that anti–HNA-3a could bind to CTL-2 expressed on endothelial cells and could induce endothelial barrier disturbance (49). In contrast, the results of our in vitro experiments suggested a minor role of endothelial CD36 in anti-CD36–mediated TRALI. First, we found low CD36 expression on the surface of HLMVECs. Second, direct priming with anti-CD36 sera did not alter the permeability and resistance of HLMVECs. However, we found that priming of monocytes with anti-CD36 Abs caused TNF-α secretion in the supernatant and upregulation of CD36 expression on monocytes when monocytes interacted with TNF-α–activated endothelial cells. Incubation of HLMVECs with TNF-α–rich supernatants from anti-CD36–primed monocytes increased endothelial permeability and decreased endothelial resistance, a finding that supports the idea of an important pathogenic role for monocytes.

Increasing evidence indicates that complement may be an important mediator of lung injury in TRALI; however, this has not yet been systematically investigated (50). One recent study demonstrated that TRALI depends on the ability of the Ab Fc domain to induce complement activation (16) on the surface of endothelial cells, as shown in the murine model of anti–MHC class I–mediated TRALI (51). Accordingly, we found that the F(ab′)_2_ fragments of mAb GZ1 did not induce TRALI, and depletion of complement or inhibition of C5 completely prevented the occurrence of anti-CD36–mediated TRALI. The role of C5 may explain the observation that anti-CD36 did not cause TRALI in female mice, which is in agreement with reports in the literature (29); female mice had much lower plasma C5 levels than did male mice. However, studies showed that estrogen could suppress lung inflammatory responses through an effect on vascular cell adhesion molecules and pro-inflammatory mediators (52), which might affect the development of TRALI. To reduce this possibility, only female mice without any signs of estrus were used in this study. Furthermore, we found that anti-CD36 could trigger generation of C5a, which is involved in the recruitment of several inflammatory leukocyte types (53). C5a enhanced TNF-α release by LPS-stimulated monocytes and macrophages (54, 55), and recent data showed that binding of C5a to C5aR on monocytes could lead to ROS generation and secretion of inflammatory cytokines such as IL-1β (56). In this respect, our in vitro experiment showed that anti-CD36 generated ROS production in monocytes. Strait et al. (12) found that anti–MHC I Abs bound to endothelial cells activated complement, leading to C5a production and recruitment of monocytes to the lungs of mice, resulting in endothelial cell damage. Based on our current in vitro results, this pathway might not apply for anti-CD36–mediated TRALI, because anti-CD36 did not directly alter endothelial permeability of HLMVECs, most probably due to very low CD36 expression on these cells. However, the exact contribution of anti-CD36 bound to endothelial cells needs to be further studied in vivo.

To date, specific treatment for TRALI is unavailable (4). In this study, we examined 3 different inhibitors. Administration of the competitive inhibitor GZ1 F(ab′)_2_ was only effective to prevent TRALI, but not as a post-TRALI remedy. This condition seemed different from FNAIT, in which deglycosylated GZ1 has been shown to prevent fetal death caused by anti-CD36 (20). Significant inhibition, however, was observed before TRALI onset, as well as after TRALI, in our study when NAC was administered i.v. together with inhalation of atomized NAC.

Among complement proteins, C5 plays a major role in complement-mediated inflammation (30). Therefore, anti-C5 treatments represent a favored target for the development of anti-complement drugs for different diseases (57–60). Recently, Zelek et al. (30) demonstrated that mAb BB5.1, which is specific for mouse C5, could effectively inhibit C5 activation and prevent C5a accumulation in mice. In comparison with untreated mice, we found that administration of mAb BB5.1 not only was able to prevent but also could therapeutically treat anti-CD36 murine TRALI.

**Figure 7:**
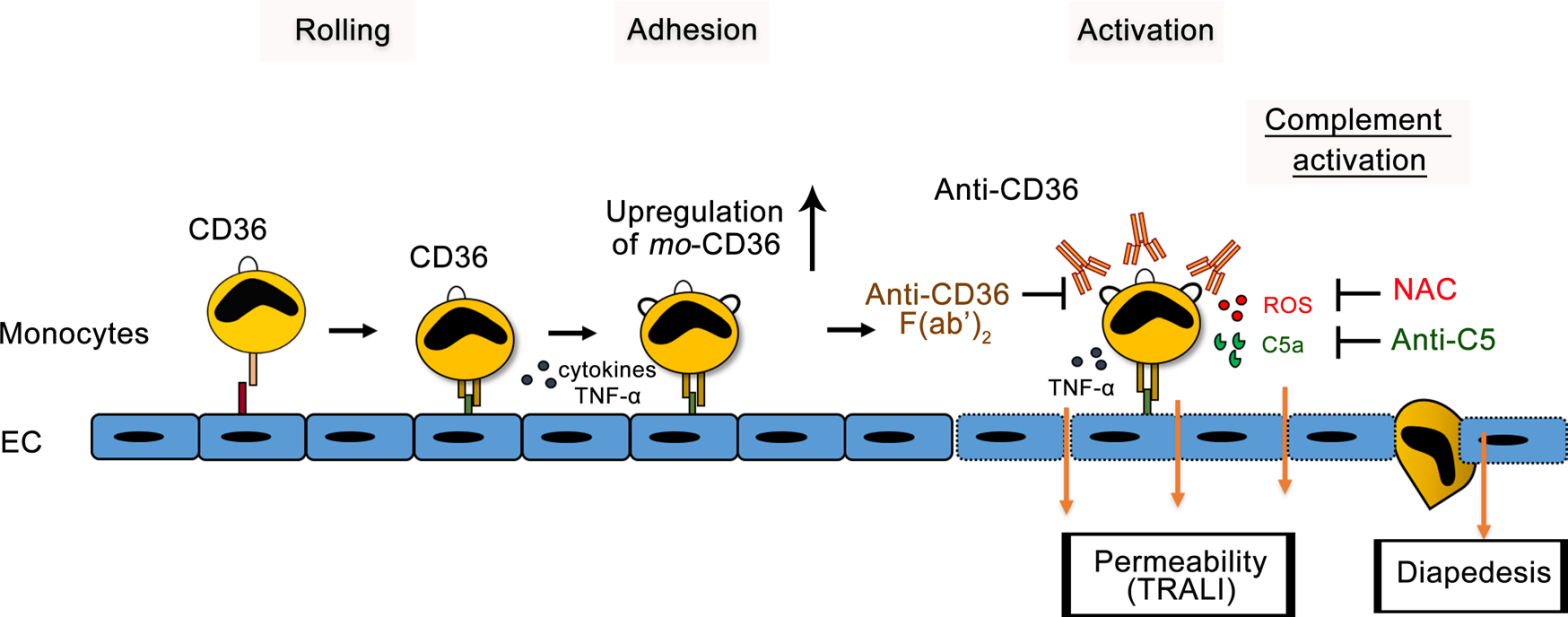
Proposed 2-hit mechanism of anti-CD36–mediated TRALI. A state of inflammation induced by a first hit (e.g., LPS) triggers the upregulation of adhesion molecules on endothelial cells (E-selectin, ICAM-1, and VCAM) and monocytes (PSGL-1 or VLA-4), leading to the rolling and firm adhesion of monocytes on an endothelial surface. Cytokine (e.g., TNF-α), which may upregulate CD36 expression on monocytes. A second hit consists of anti-CD36 Abs bound to adherent monocytes. Upregulation of CD36 on monocytes enhances Ab binding and monocyte activation. This reaction triggers complement activation (accumulation of C5a), generation of ROS, and cytokine secretion (e.g., TNF-α). This reaction cascade causes lung endothelial disturbance, strengthened by the recruitment of other blood cells (neutrophils or platelets) (not shown), and monocyte extravasation (diapedesis), resulting in severe TRALI symptoms. The potential targets for possible inhibitors — anti-CD36 F(ab′)_2_, NAC, and anti-C5 — are indicated.

Overall, our observations suggested the following mechanism and intervention of anti-CD36–mediated TRALI (Figure 7): interaction between monocytes and TNF-α–activated lung endothelial cells leads to the upregulation of CD36 expression on monocytes, resulting in increased Ab binding, triggering complement activation, ROS generation, and cytokine production, which are all responsible for the severe endothelial dysfunction in TRALI.

In conclusion, we shed light on the pathomechanism of anti-CD36–mediated TRALI using a 2-hit murine model. In this model, we prevented the occurrence of TRALI by prophylactic injection of GZ1 F(ab′)_2_, NAC, or mAb BB5.1. More interestingly, we also achieved a good therapeutic effect of mAb BB5.1 on established anti-CD36–mediated TRALI. The potential for effective therapy reinforces the need for increased awareness of anti-CD36–induced TRALI, particularly in Asian populations. Although some therapeutic approaches are suggested by our work, more research on the detailed mechanism with sera from different patients with TRALI should be performed to expand the relevance of these findings.

## Methods

### Mice.

WT C57BL/6J mice were purchased from the Laboratory Animal Centre of the Sun Yat-Sen University. *Cd36^–/–^* mice were purchased from Jackson Laboratory (*B6.129S1-Cd36^tm1Mfe^/J*). All mice, aged 8–10 weeks, were housed under specific pathogen–free conditions.

### Abs against CD36.

Mouse mAbs against CD36 were generated as previously described (20). One mAb, termed GZ1 (IgG2a), was selected for this study. Human anti-CD36 sera samples were collected from patients with FNAIT (21), platelet transfusion refractoriness (19), and TRALI (23) (anti-CD36 1, 2, 3, respectively). Anti-CD36 1 and 2 were characterized in our Guangzhou Blood Centre in Guangzhou, and anti-CD36 3 was a gift from the Japanese Red Cross. IgG was purified using Melon Gel IgG Purification Kits (Thermo Fisher Scientific).

### Murine TRALI models.

Male and female WT C57BL/6J mice (*Cd36*^+/+^) and *Cd36^–/–^* male mice were untreated or pretreated (2-hit model) with LPS (0.1 mg/kg *Escherichia coli* O111:B4, Sigma-Aldrich) i.p. 24 hours before i.v. injection with 400 μL of the mAb GZ1 (0.4 mg/kg) or GZ1 F(ab′)_2_ (0.8 mg/kg) (13). IgG2a isotype (0.4 mg/kg, i.v.; clone C1.18.4, Bio X Cell) was used as a control. In some experiments, 400 μL (5.6 mg) of purified IgG isolated from human sera was administered. Rectal temperatures were measured at 30 minutes after Ab injection using a digital thermometer (Yuyan). Mice were euthanized by i.p. injection of sodium pentobarbital at 2 hours after anti-CD36 administration (9). The lungs were harvested and lung W/D weight ratios were determined.

### Pao_2_ and Sao_2_.

Using a handheld blood analyzer (i-STAT 1, Abbott), the Pao_2_ and Sao_2_ were measured in blood samples collected in a syringe containing heparin anticoagulant (50 U/mL) from the abdominal aortas of the anesthetized mice at 10 minutes after anti-CD36 injection, using an i-STAT CG4^+^ cartridge.

### Analysis of BALs.

BALs were performed as previously described (9), with minor modifications. The BAL was performed at 30 minutes after administration of anti-CD36 or the isotype control. The protein contents of BALs were quantified using a bicinchoninic acid assay using BSA as a standard (Thermo Fisher Scientific). CXCL2/MIP-2, TNF-α, and KC concentrations were measured by ELISA (Quantikine ELISA Kit, R&D Systems).

### Lung W/D weight ratios.

The W/D weight ratio of the lungs of mice was defined as a parameter of pulmonary edema. At 2 hours after anti-CD36 infusion, mice were anesthetized using 2, 2, 2-tribromoethanol (Avertin) i.p. (2% final in PBS), and the chest cavity was exposed. The whole lung was removed, weighed, dried in an oven at 60°C for 72 hours, and reweighed. The lung W/D weight ratio was calculated by the following formula: net wet weight/net dry weight.

### Histology.

At 2 hours after TRALI induction with anti-CD36, mice lungs were removed and fixed overnight with 4% paraformaldehyde (Sigma-Aldrich). Lung sections (8 μm) were stained with H&E and examined with a Leica DMI3000 B inverted microscope.

### In vivo depletion of monocytes, neutrophils, platelets, and complement.

Monocytes were depleted using clodronate liposomes, as previously described (13). Clodronate-liposomes or PBS-encapsulated liposomes (clodronate 5 mg/mL, Liposoma BV, 50 mg/kg BW) were administered by tail vein injection (i.v.) for 6 hours. The rate of monocyte depletion in the blood was examined before and after depletion by flow cytometry using FITC-conjugated CD11b (clone M1/70, BD Pharmingen) and APC-conjugated F4/80 (clone M8.1, Biogems) markers as recommended (41). Platelets were depleted by i.v. administration of anti–GPIb-α Abs (2 mg/kg; R300, Emfret Analytics) 24 hours before TRALI induction, as previously described (37). Isotype IgG (2 mg/kg; C301, Emfret Analytics) was used as a control. The platelet count was measured using an animal automatic hematology analyzer (BC-5000 Vet, Mindray). Neutrophils were depleted by i.p. injection of 20 mg of hydroxyurea (Sigma-Aldrich) for 7 days and subsequently by i.v. injection of anti-Ly6G mAb (5 mg/kg; clone 1A8, Bio X Cell) 48 hours and 24 hours before TRALI induction, respectively (12). Neutrophil depletion was monitored by cell counting and verified by flow cytometry using PE-Cy7–conjugated CD45 (clone 30-F11, BD Pharmingen), FITC-conjugated CD11b (clone M1/70), and PE-conjugated CD115 (clone T38-320, BD Pharmingen) markers, as previously described (13). Complement was depleted by i.p. injection of cobra (*Naja kaouthia*) venom factor (0.4 mg/kg i.p.; catalog 233552, MilliporeSigma) or PBS as a control 24 hours before TRALI induction (51). The level of complement factors was detected by C5 ELISA to verify the efficiency of complement depletion (Supplemental Figure 2E).

### Complement ELISAs.

Whole blood samples were collected from mice 2 hours after the infusion with mAb GZ1 or LPS, for controls. Naive mice were treated as the control. Serum was isolated after blood coagulation on ice for 2 hours and stored at –80°C until use. ELISAs for mouse C3 (catalog D721061, Sangon Biotech), C5 (catalog ab264609, Abcam), and C5a (catalog D721063, Sangon Biotech) were used according to manufacturers’ instructions.

### Plasma transfer.

Plasma transfusion was performed as previously described (12) with minor modification. Blood samples were collected from the tail veins of male mice into 3.2% sodium citrate–containing tubes (1:10 dilution) at 4°C. Plasma was separated, pooled, and stored at –80°C. Heat-inactivated pooled plasma (56°C for 30 minutes) was used as the control. The mAb GZ1 or isotype control was diluted in 300 μL of the pooled plasma for TRALI induction, as described above.

### Preparation of F(ab′)_2_ fragments.

F(ab′)_2_ fragments were generated as previously described (41). The purity of GZ1 F(ab′)_2_ fragments was determined by silver staining of proteins during SDS-PAGE and confirmed by flow cytometry using fluorescently labeled secondary Abs specific for Fc-fragments (1:200; Jackson ImmunoResearch) or against heavy and light chains (1:200; Thermo Fisher Scientific) (Supplemental Figure 4)

### Detection of mAb GZ1 and human serum containing anti-CD36 reacting with mouse platelets by flow cytometry.

Mouse and human anti-CD36 were assessed by flow cytometry with platelets from *Cd36^+/+^* or *Cd36^–/–^* mice as previously described (20). MAb GZ1 (0.25 μg) and human anti-CD36 sera 1, 2, and 3 (dilution, 1:16) were incubated with 100 μL of EDTA-treated blood (1:100) from *Cd36^+/+^* or *Cd36^–/–^* mice for 30 minutes at room temperature and washed with PBS/1% BSA. IgG2a isotype control and human AB normal serum samples were treated as negative controls. We then added 50 μL of FITC-conjugated anti-mouse IgG or anti-human IgG (1:200; Jackson ImmunoResearch) for 30 minutes. After RBC lysis with lysing buffer (BD Biosciences), cells were resuspended in 0.5 mL PBS/1% BSA and then analyzed by flow cytometry (FACS Canto II, BD Biosciences).

### Measurement of cytokine secretion in vitro.

PBMCs were isolated from healthy CD36-positive blood donors using Ficoll-Paque premium (GE Healthcare). PBMCs were primed with anti-CD36, and the culture supernatant was analyzed for cytokine secretion as described in initial studies (61). Briefly, human anti-CD36 3 (20%, vol/vol) was incubated with *Cd36^+/+^* PBMCs for 20 hours at 37°C, and then the culture supernatant was collected, centrifuged at 13,000*g* for 5 minutes in 4°C, and stored at –80°C until use. AB serum samples from healthy individuals were treated as the control. The concentration of TNF-α in the supernatant was measured by solid phase ELISA (R&D Systems).

### Endothelial permeability assay.

A permeability assay was performed as described previously with minor modifications (62). Primary HLMVECs (catalog 3000, Sciencell) were maintained in endothelial cell medium (Sciencell). Endothelial cells at passage 5 were then plated onto Transwells (6.5 mm diameter, 0.4 mm pore size; Corning) at a density of 10^5^ cells/insert precoated with 50 μg/mL fibronectin (Sigma-Aldrich) for 1 hour at 37°C. HLMVECs were cultured for 2 days to achieve a monolayer and then treated for 6 hours with PBMC supernatant diluted (1:1) with culture medium containing 0.25 U/mL heparin (Ratiopharm). Endothelial permeability was measured by the migration of FITC-labeled albumin (400 μg/mL, 66 kDa; Thermo Fisher Scientific) for 2 hours using a fluorescent plate reader (FLX800, Bio-Tek) at an excitation of 485 nm and emission of 538 nm.

### TEER assay.

Aliquots of HLMVECs (10^5^ cells) were cultured on Transwells as described above and then connected to an electrical cell substrate impedance system (cellZscope, nanoAnalytics). After adding 50 μL of supernatant from PBMCs diluted in 210 μL of culture medium (apical), the electrical resistance was recorded for 24 hours at 37°C in a cell incubator. The basolateral area contained 810 μL of culture medium.

### Measurement of ROS production by flow cytometry.

PBMCs (0.5 × 10^6^ cells/well) were seeded in 24-well plates and stained with 20 μM 2,7-dichlorodihydrofluoroscein diacetate (Abcam) solution for 30 minutes at 37°C in the dark, as previously described (63). Subsequently, these PBMCs were stimulated with mAb GZ1 (10 μg/mL) for 20 hours or tert-butyl hydroperoxide (55 μM) as a control. In some experiments, PBMCs were incubated with 30 mM NAC (Sigma-Aldrich) for 30 minutes before stimulation with mAb GZ1. Cells were then gently pipetted to obtain single-cell suspension; monocytes were gated using side scatter versus forward scatter plots and analyzed by flow cytometry (BD FACSCanto II).

### RNA isolation and RT-PCR.

Monocytes and HLMVECs were incubated with medium alone or in medium containing TNF-α (10 ng/mL) for 4 hours, as previously described (64). Total RNA of monocytes and HLMVECs was isolated using the Direct-Zol RNA Miniprep kit after treatment with DNAse (Zymo Research) (65). Aliquots of RNA (1 μg) were reverse-transcribed using the Transcriptor First Strand cDNA Synthesis kit (Roche). PCR assays for CD36 and β-actin cDNA were performed using TB Green Premix Ex Taq II (Tli RNase H Plus, TaKaRa) on a 7500 Fast Real-Time PCR System (Applied Biosystems). The cycling program consisted of 1 denaturation cycle of 30 seconds at 95°C, followed by 40 cycles of 5 seconds at 95°C and 34 seconds at 60°C. The primer was 5′-TGTAACCCAGGACGCTGAGG-3′, and the reverse primer was 5′-GAAGGTTCGAAGATGGCACC-3′ for CD36; the primer and reverse primer were 5′-CCTCACCCTGAAGTACCCCA-3′ and 5′-TGCCAGATTTTCTCCATGTCG-3′, respectively, for β-actin.

### Endothelial-monocyte co-culture assay.

HLMVEC co-culturing with monocytes was performed as previously described (66). Monocytes (purity > 90%) were isolated from PBMCs using the EasySep Human Monocyte Isolation Kit (STEMCELL Technologies). Monocytes (10^6^ cells) were then added to untreated or TNF-α–treated HLMVECs for 4 hours at 37°C. Cells were solubilized using RIPA buffer (Thermo Fisher Scientific) and analyzed by immunoblotting, as previously described (67). Aliquots of 15 μg of protein per lane were separated by 10% SDS-PAGE under reducing conditions, transferred onto PVDF membranes (Millipore), and incubated with mAbs against CD36 (clone D8L9T, Cell Signaling Technology), or CD14 (clone 1H5D8, Abcam), or β-actin (clone 8H10D10, Cell Signaling Technology) overnight at 4°C. The membranes were then washed and stained with HRP-labeled goat anti-rabbit Ab (dilution 1:3,000; Cell Signaling Technology) or donkey anti-mouse Abs (dilution, 1:3,000; Jackson ImmunoResearch). Bound Abs were detected using ECL substrates (Bio-Rad). CD36, CD14, and β-actin bands were quantified by Volume Box Tools using Image Lab software (Bio-Rad).

### Administration of anti-FcγR inhibitor and IVIG.

Rat IgG2b anti–FcγRII/III mAb (clone 2.4G2, Bio X Cell) and human IVIG (Boya Bio-Pharmaceutical) were administered i.p. to the mice at a dose of 25 mg/kg or 2 g/kg at 24 or 18 hours, respectively, before TRALI induction. The rat IgG2b isotype control (clone LTF-2, Bio X Cell) and human serum albumin (Sigma-Aldrich) were injected as controls.

### Therapeutic intervention of TRALI induced by anti-CD36.

Three inhibitors — mAb GZ1 F(ab′)_2_ (41), NAC (12), and the neutralizing Ab mAb BB5.1 — against mouse C5 complement (30) were administered after TRALI induction with 200 μL of mAb GZ1 (0.4 mg/kg). Precisely, 200 μL of mAb GZ1 F(ab′)_2_ (5 mg/kg in PBS), 200 μL of BB5.1 (1 mg/200 μL in PBS), or 200 μL of NAC solution (10 mg in saline; Sigma) was administered 30 or 5 minutes before TRALI was induced (prophylactic treatment). In addition, each inhibitor was injected at the dose after TRALI was induced (therapeutic treatment) 3 minutes after the rectal temperature dropped 0.5°C, which we considered the onset of the TRALI (Supplemental Figure 5). For the therapeutic treatment with NAC, besides injection of NAC solution, aerosolized NAC was given for 20 minutes after NAC injection in an inhalation chamber. The nebulizer (NE-C25S, Omron) in the inhalation chamber was operated under the following conditions: air flow at least 8 L/min, nebulization rate at least 0.2 mL/min, and nebulizer flow 4 L/min. Aerosolized NAC solution (100 mg/mL) was from Shuyaqi (Guorun Pharma) and was spray-dried (aerodynamic diameter < 5.0 μm).

### Statistics.

Data, presented in this study as the mean ± SD, were analyzed using GraphPad Prism 6.0. Comparisons between 2 groups were assessed using an unpaired Student’s *t* test (2-tailed). Statistical analysis was performed with 1-way ANOVA with Bonferroni’s correction for multiple comparisons. Kaplan-Meier methods were used to estimate survival durations, and comparison between the 2 subgroups was done using a log-rank test. *P* < 0.05 was considered statistically significant.

### Study approval.

The Animal Care Committee of Sun Yat-Sen University, Guangzhou, China, approved this study (no. SYSU-IACUC-2020-000185).

## Author contributions

DWC, YSF, SS, and RK conceived and designed research; DWC, TK, XZX, WJX, YBW, YRX, JL, JD, YKC, HQD, HR, and MA performed experiments; DWC, TK, XZX, XY, WMZ, BPM, SS, YSF, and RK analyzed data; DWC, TK, XZX, YSF, and SS wrote the manuscript and RK analyzed and revised the manuscript.

## Supplementary Material

Supplemental data

## Version 1 02/21/2023

In-Press Preview

## Version 2 03/22/2023

Electronic publication
