# Supplementary material for: Mechanism and intervention of murine transfusion-related acute lung injury caused by anti-CD36 antibodies
Source: JCI Insight. 2023 Mar 22;8(6):e165142. doi: 10.1172/jci.insight.165142 (PMC10070104; doi:10.1172/jci.insight.165142)

Supplemental Figure 1: Anti-CD16/CD32 or IVIG prophylactical treatment could prevent mice from anti-CD36 induced TRALI.

(A) Rectal temperatures and (B) lung W/D weight ratios of LPS pre-treated male mice first prophylactically treated with anti-CD16/CD32 or IVIG, then mAb GZ1 IgG were administered. Isotype IgG2b or HSA was treated as control. Statistical analysis was performed with 1-way ANOVA with Bonferroni's correction for multiple comparisons. Each dot represents one mouse (n = 5 in each group) and error bars represent the SD. ****p<0.0001, *ns*: non-significant.

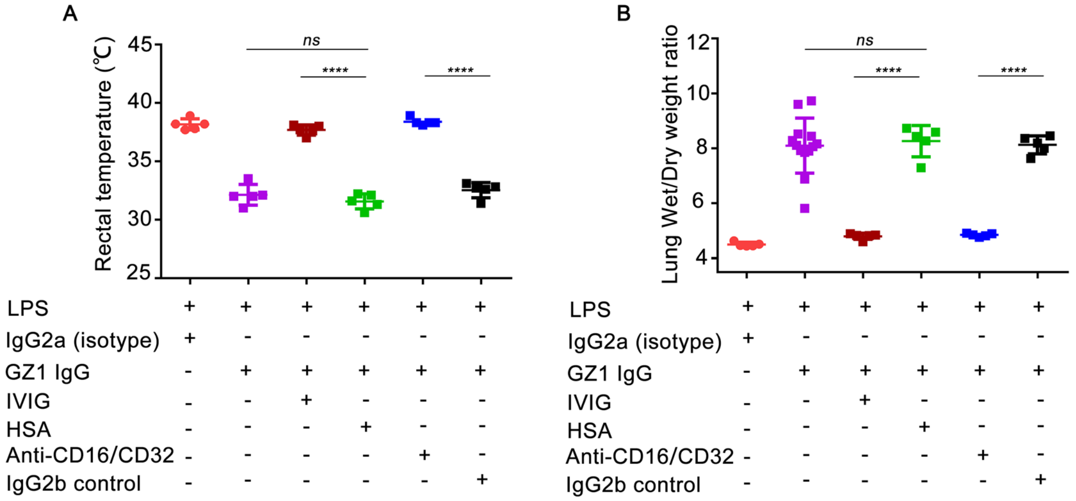

Supplemental Figure 2: Analysis of monocytes, neutrophils, platelets and complement depletion efficiency.

(A) Representative flow cytometric analysis of peripheral blood monocytes before and after depletion with clodronate-liposome are shown. Monocytes were stained with mAbs against CD11b (FITC) and F4/80 (APC). (B - C) Analysis of neutrophil population before and after depletion with hydroxyurea and mAb anti-ly6G (1A8) treatment. Neutrophil was stained with mAbs against CD45 (PE-Cy7), CD11b (FITC) and CD115 (PE) by flow cytometry test (B) and counted by animal automatic haematology analyser (C, n = 20). (D) Platelet count was determined by animal automatic haematology analyser before and after depletion with mAb against GPIbα (n = 20). (E) Concentration of C5 in male mice was measured without or with treatment of cobra venom factor (CVF) (n = 5). Statistical analysis was performed with a two-tailed unpaired Student's *t*-test. Each dot represents one mouse and error bars represent the SD. ****$p<0.0001$.

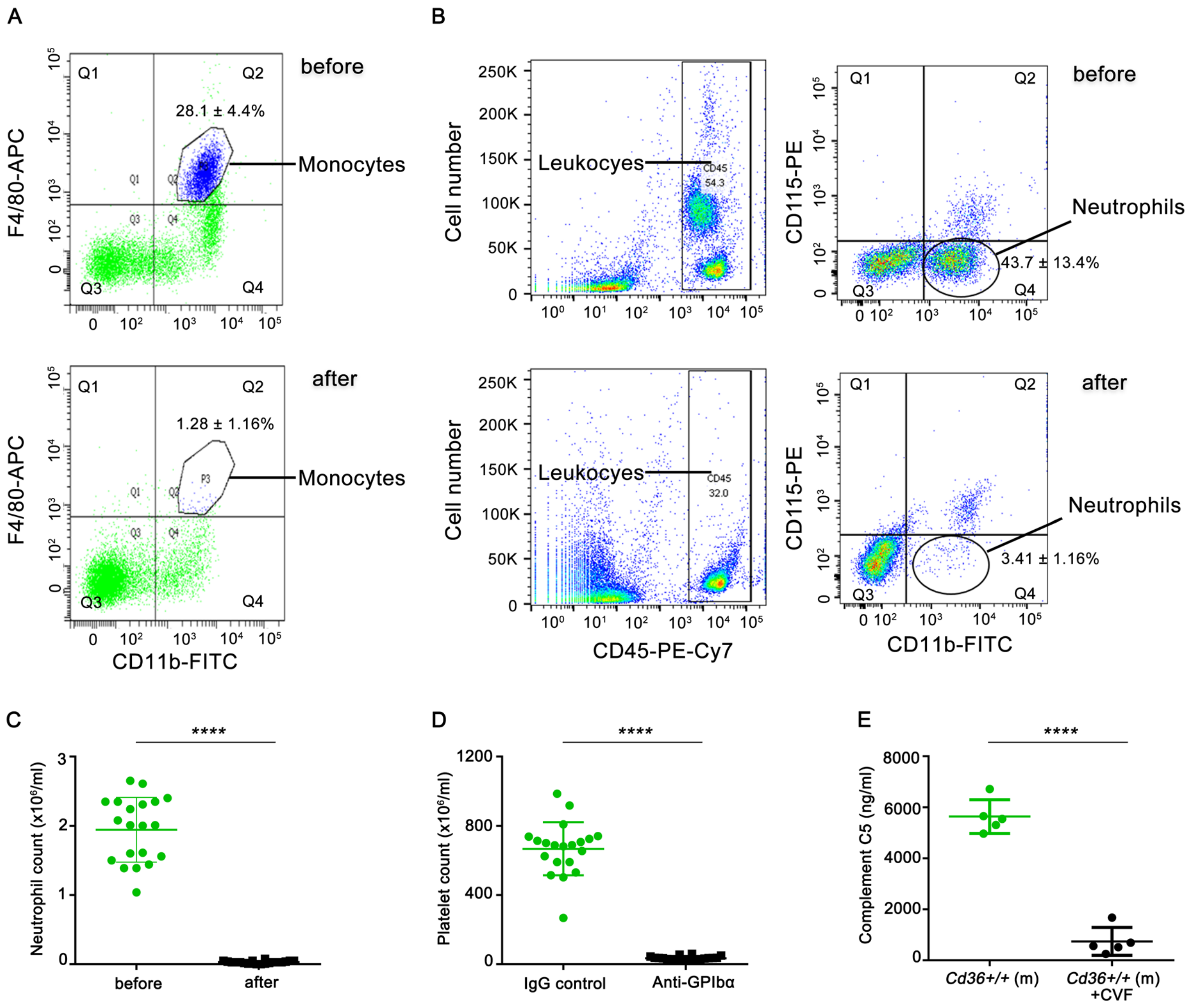

Supplemental Figure 3: Female mice transfused with plasma from male mice induced TRALI by anti-CD36 administration.

(A) Concentration of protein in bronchoalveolar lavage of LPS pre-treated *Cd*36+/+ female mice untreated or treated with plasma from male mice diluted mAb GZ1, IgG2a isotype were treated as control. (B) Histology performed on lung tissue from the indicated female mice groups. Lung tissue sections were stained with hematoxylin and eosin *(H&E)* and images were taken at 20x magnification. Representative images from each indicated group are shown. Scale bars represent 100 μm. Statistical analysis was performed with 1-way ANOVA with Bonferroni's correction for multiple comparisons. Each dot represents one mouse (n = 5 mice in each group) and error bars represent the SD. ****p<0.0001, *ns*: non-significant.

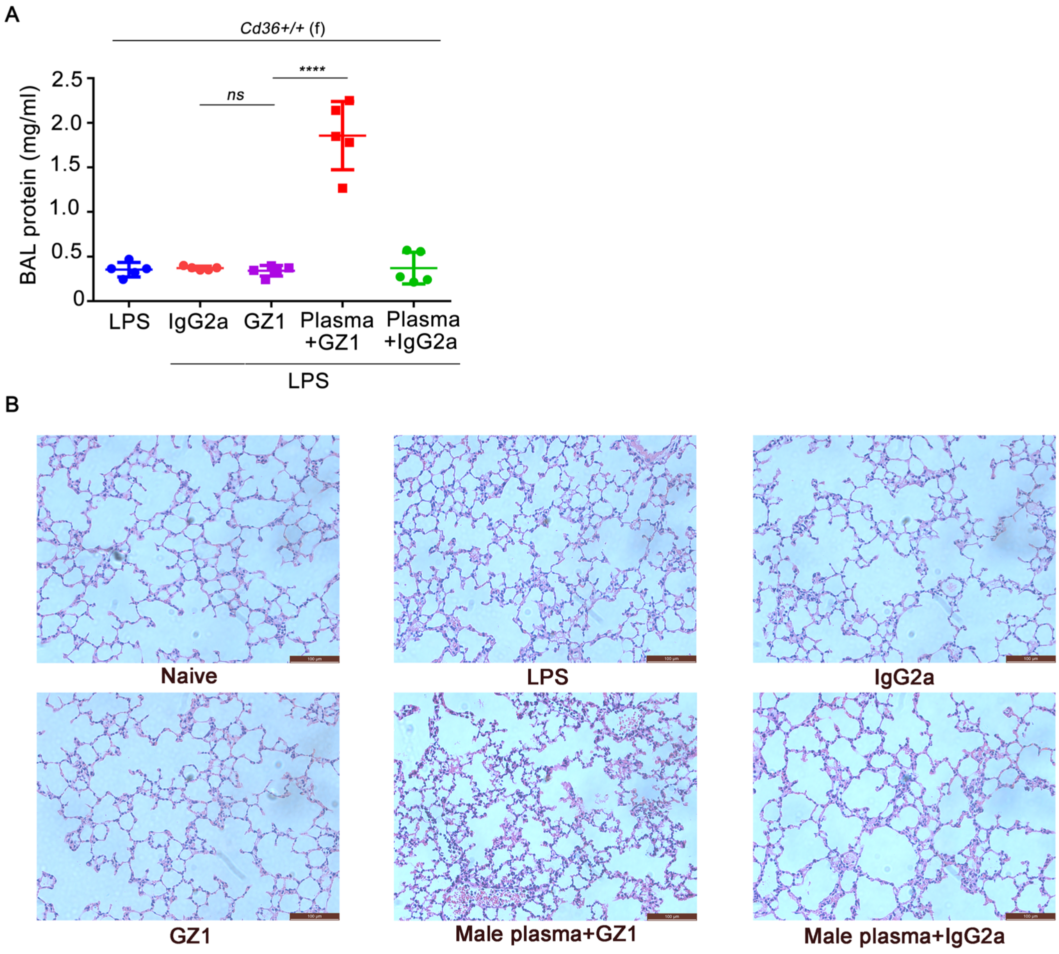

Supplemental Figure 4: Analysis of F(ab')$_2$ fragments of mAb GZ1 by silver staining and flow cytometry.

Panel A: purified IgG and F(ab')$_2$ fragments for mAb GZ1 were run on 7.5% SDS-PAGE gels under nonreducing (NR) and reducing (R) conditions. Separated proteins were visualized by silver staining. *Panel B*: platelets from wild-type mice were incubated with purified IgG or F(ab')$_2$ fragments from mAb GZ1. After washing, bound antibodies were detected with Fc-specific FITC conjugated goat anti-mouse IgG or H+L specific Alexa Fluor 488 labelled donkey anti-mouse IgG.

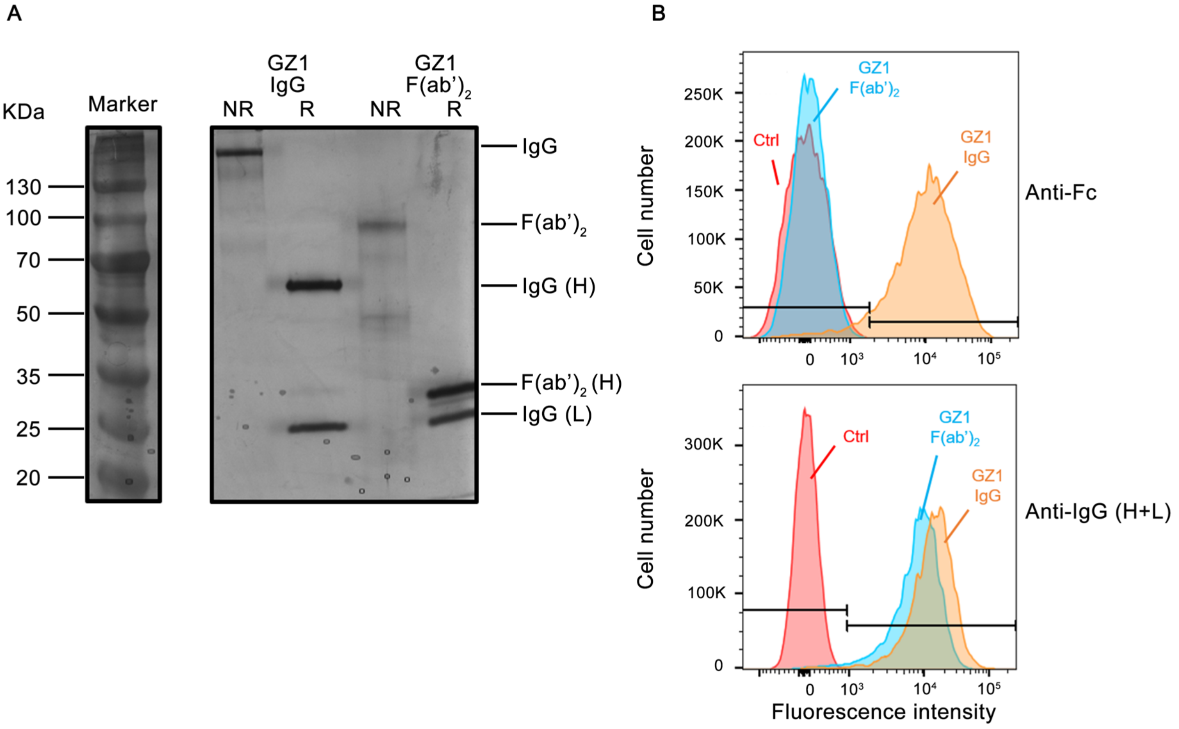

Supplemental Figure 5: Drop of rectal temperature in three min after TRALI induction.

LPS pre-treated *Cd*36+/+ male mice were treated with mAb GZ1 (0.4 mg/kg) or isotype IgG2a (0.4 mg/kg), respectively. Rectal temperatures were measured three min after antibody injection. Statistical analysis was performed using a two-tailed unpaired Student's *t*-test. Each dot represents one mouse (n = 6 in each group) and error bars represent the SD. $^{**}p < 0.01$.

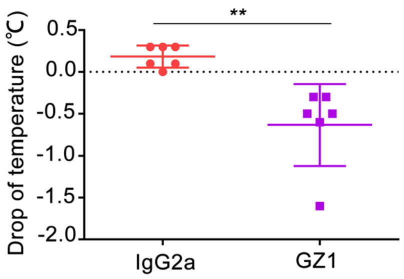

Figure 1 for the Reviewer: Analysis of mice whole blood derived from C*d*36+/+ and C*d*36-/- by flow cytometry.

A) Monocytes/macrophages from C*d*36+/+ and C*d*36-/- mice were identified using FITC-labelled anti-CD11b. Subsequently, CD11b positive cells were double stained with PE-labelled anti-F4/80 and APC-conjugated anti-CD36. CD36 positive monocytes were presented in C*d*36+/+ mice only, but not in C*d*36-/- mice.

B) Platelets were stained with APC-conjugated anti-CD36. CD36 positive platelets were only detected in C*d*36+/+ mice.

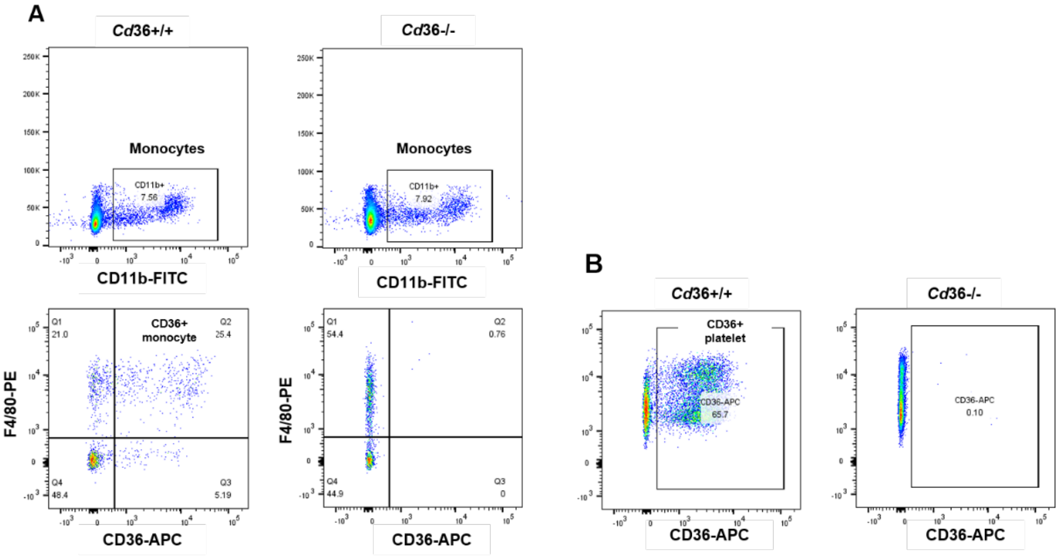

Figure 2 for the Reviewer: Analysis of PBMC fraction adherent to the plate by flow cytometry.

For the ROS assay, we used PBMC fraction adherent to the microtiter plate, which are predominantly monocytes (CD14+).

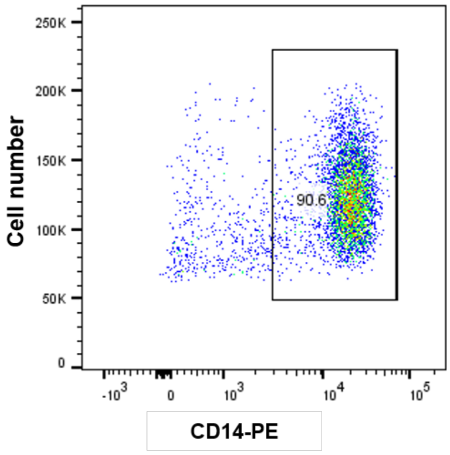

Figure 3 for the Reviewer: Analysis of different dose of anti-CD36 induced TRALI in C*d*36+/+ male mice.

(A) Rectal temperatures and (B) lung W/D weight ratios of mice first treated with LPS, then different dose of mAb GZ1 IgG were administered. Statistical analysis was performed with 1-way ANOVA with Bonferroni's correction for multiple comparisons. Each dot represents one mouse (n = 5 in each group) and error bars represent the SD. ****p<0.0001, ***p<0.001, *ns*: non-significant.

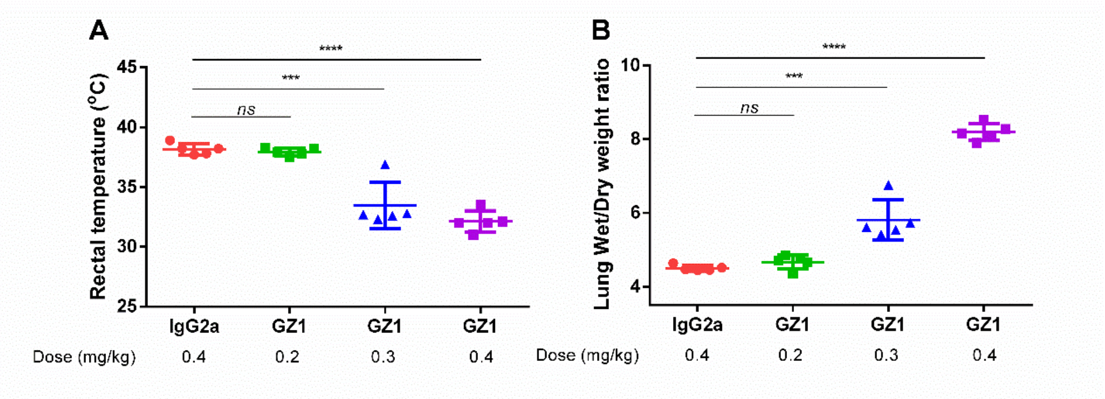

Supplement: Supplemental data [file jciinsight-8-165142-s038.pdf]
